# Coats of Variable Hue in Tunisian Hares (Genus *Lepus*): Population Genetics and Mitochondrial Phylogenetics for Species Delimitation

**DOI:** 10.3390/ani16081236

**Published:** 2026-04-17

**Authors:** Asma Awadi, Hichem Ben Slimen, Felix Knauer, Milomir Stefanović, Franz Suchentrunk

**Affiliations:** 1 Laboratory of Functional Physiology and Valorization of Bioresources, Higher Institute of Biotechnology of Béja, University of Jendouba, Béja 9000, Tunisia; awadiasma@gmail.com (A.A.); hichem.benslimen@isbb.rnu.tn (H.B.S.); 2 Research Institute of Wildlife Ecology, University of Veterinary Medicine Vienna, Savoyenstraße 1, A-1160 Vienna, Austria; felix.knauer@vetmeduni.ac.at; 3 Department of Biology and Ecology, Faculty of Sciences, University of Novi Sad, 21000 Novi Sad, Serbia; milomir.stefanovic@dbe.uns.ac.rs; 4 Wildlife Research—Vienna & Bredasdorp, Bredasdorp 7280, South Africa

**Keywords:** hares, North Africa, coat colors, phylogenetics, gene flow, microsatellites, mtDNA

## Abstract

In Africa, hares (*Lepus* spp.) show a high variability in external phenotypes. Species identification, delimitation, and distinction are difficult, due to the generally shallow evolutionary divergence of species, high intraspecific phenotypic variability, potentially complex reticulate evolutionary scenarios, and absence of relevant data across large areas. Our microsatellite and mitochondrial (mt) CR1 sequence data of hares from Tunisia with four different coat color morphs revealed high levels of gene flow both in nuclear and mt gene pools but no relevant partitioning of genetic variability parallel to the four coat color types. Nuclear and mt gene pool correspondence was at a very low level, and no phylogenetic gaps were observed in the mtDNA. Our results indicate only one hare species with different coat colors in Tunisia that shows no signals of reticulate evolution or incipient speciation. Because of potential reticulate evolutionary signals, especially in the mtDNA and the probably high adaptive significance of coat colors and patterns, external phenotypes combined with mtDNA alone may not serve as good proxies for species delimitation in hares from wide ranges in Africa. Combined nuclear and mtDNA population genetic and phylogenetic data allow more complex inferences on current and ancestral evolutionary processes for species identification and delimitation.

## Simple Summary

In the genus *Lepus*, reticulate evolutionary processes can obscure species boundaries, especially when range-wide or robust and meaningful phenotypic and genetic data are lacking. We tested several genetic hypotheses for Tunisian hares with four different color morphs using population genetic and phylogenetic methods. Although genetic diversity was high, the results consistently showed extensive gene flow among regions and morphs. Mitochondrial (mt) lineages revealed no wide phylogenetic gaps that would signal ancestral introgression from other species or shared ancestral polymorphism from incomplete lineage sorting. Also, no signals of incipient speciation were found. All findings contradicted our alternative hypotheses and accorded to our null hypothesis of a single species. Nevertheless, to prevent potential unnecessary taxonomic confusion in the future, we refrain from a definitive species assignment for Tunisian hares until broader African genetic data are available, specifically including the diverse forms traditionally considered belonging to *L. capensis*, *L. saxatilis*, and *L. victoriae*. Ideally, species delineation within the genus *Lepus* should be based on a biological or genetic species concept, which requires explicit information on successful reproduction and nuclear gene flow or/and genome data collected from comprehensive and geographically representative samples, rather than basing species assignment on (external) phenotypes and mtDNA alone.

## 1. Introduction

Hares and jackrabbits (*Lepus* spp.; Leporidae, Lagomorpha) exemplify a young evolutionary radiation, e.g., refs. [1,2,3,4], with adaptations to many environmental conditions. Native or introduced populations occur in semi-arid grasslands, in hot and cold arid regions, in Mediterranean-type environments, temperate open or semi-open landscapes, as well as in arctic–alpine and high-altitude habitats and in various types of agricultural and pastoral land in most regions of the world, but are less common or absent from dense forests, e.g., ref. [5].

In addition to the large intraspecific variability and big interspecific overlap of body and skull proportions and minor skeletal or dental characters, hares exhibit a great deal of variability in coat colors and patterns—e.g., refs. [6,7,8,9,10]. This high intra- and interspecific variability has traditionally made it difficult to identify species status and associated boundaries for many *Lepus* forms, especially when only few or no specimens were available from larger regions with different ecological conditions between studied forms and for allopatric occurrences.

Historically, many forms were described as separate *Lepus* species solely based on external phenotypes, i.e., coat characteristics, simple morphometric proportions, and minor dental and skeletal characters [11,12,13,14], but many of them were later considered synonyms and grouped into fewer species—e.g., refs. [5,15] (also, for synonyms).

Meanwhile, biochemical and molecular genetic as well as genomic approaches have greatly advanced our understanding of evolution and systematics of hares, while also revealing significant evolutionary dynamics throughout both the early history of this genus and its later development into diverging species. Many of these studies traced reticulate evolutionary trajectories in the genus, e.g., refs. [16,17,18,19], instead of a strictly anagenetic pattern of gene pool differentiation following cladogenetic periods leading to genetically distinct species, i.e., with effective (pre- and postzygotic) barriers to gene flow or only marginal gene exchange but the potential for continuous separate evolutionary differentiation. These reticulate evolutionary patterns include evolutionary recent (shallow) cladogenetic divergence and concomitant gene flow during speciation with retained ancestral polymorphism, recurrent symmetric or asymmetric introgression both in mitochondrial (mt), and nuclear genomes after hybridization upon secondary contact with introgression levels varying across regions—e.g., refs. [20,21,22,23,24]. Also, reduced female reproductive success due to males carrying divergent (non-native) mtDNA (inherited by females), i.e., the “Mother’s Curse effect” [25,26] has been observed, which may aggravate intraspecific mt gene pool differentiation in combination with female philopatry. Moreover, positive selection on mtOXPHOS genes [27,28,29,30] may also affect systematic inferences when they are based predominantly on mtDNA. Finally, signals of selective sweeps in nuclear genomes, e.g., refs. [31,32] can make the interpretation of species boundaries difficult, especially when based on few genes that reflect mainly (ecological) adaptation.

Operationally, such complicated evolutionary scenarios and spatially varying introgressive hybridization can in principle be addressed by genomic approaches, e.g., refs. [33,34], which also can identify genome compartments of restricted gene flow. But even pronounced species-specific genetic differences in essential cellular functions—such as growth, cell cycle regulation, respiration, and metabolism—do not necessarily appear to act as strict (postzygotic) barriers to gene flow between “good *Lepus* species”, i.e., traditionally and commonly accepted species, such as brown hares, *L. europaeus*, and mountain hares, *L. timidus* [5,15,34]. Pronounced genomic divergence may not necessarily justify species splitting in leporids. For example, the European wild rabbit, *Oryctolagus cuniculus*, comprises two different genome forms, *O. c. cuniculus* and *O. c. algirus*, with markedly reduced gene flow in genomic islands and strong differentiation on parts of the X chromosome [35], but these divergent genomic forms continue to be classified as conspecific—e.g., [32,36] (and literature cited therein). In fact, genomic data indicating islands of restricted or interrupted gene flow may result in an artificial/false distinction of species (“over splitting”), if intraspecific population genetic patterns of differentiation are not appropriately considered, such as under the “multispecies coalescent model” [37].

For the genus *Lepus*, traditional population genetic analyses using microsatellites have often provided useful information on paternity, gene exchange between nuclear gene pools, and their cohesion or population differentiation across larger regions, or have contributed to the detection of introgressive hybridization between species, independent of or in addition to classical phylogenetic approaches based on mtDNA sequences—e.g., refs. [28,38,39,40,41].

In North Africa, several forms of hares were described earlier as separate species different from cape hares, *L. capensis*, primarily based on their coat color, body, and skull size and proportions, as well as dental characters—e.g., refs. [42,43,44,45,46,47]. Later, however, all these forms were considered conspecific with *Lepus capensis* L., 1758, except for hares from an isolated population (around Beni Abbes) in western Algeria, which, mainly based on the shape of the enamel groove of the upper principal incisors, were classified as African savannah hares, *L. victoriae*—e.g., ref. [5].

More recently, however, this taxonomic concept has been questioned again, e.g., ref. [15], and additional phenotypic and genetic data have led to the splitting of forms that were previously included in *L. capensis*. For example, *L. capensis schlumbergeri* from the Spanish territories of Ceuta and Melilla in North Africa [48] were later considered *L. schlumbergeri* in the Spanish Mammal Atlas, 2007 [49], and two forms, *L. saharae* [50] and *L. berbericus* [51], were described recently as new species, the former as an extant species and the latter as an extinct species from the late Pleistocene. *L. capensis mediterraneus* and *L. c. schlumbergeri* have recently been considered separate species [50]. However, preliminary phylogenetic, phylogeographic, and population genetic data were not conclusive about the systematic status of North African hares: populations in the western Sahara/Sahel zone showed a relatively high gene flow despite high mt lineage divergence [52], leaving open the possibility of the presence of “cryptic species”.

In this study, we combined the analyses of microsatellites and mt CR1 sequences for population genetic and phylogenetic conclusions about whether the hares with four different coat colors observed in our Tunisian study area [53] represented a single biological/genetic species, incipient speciation, or two or more different species; with our approach, we also considered the possibility of signals of reticulate evolution, as in East African species [54], or of cryptic speciation.

Specifically, we tested for deviations from our null hypothesis (H_0_) that the species represents a single panmictic population—or only weakly differentiated populations—with largely unrestricted nuclear gene flow and minimal spatial structure in the mitochondrial gene pool. We contrasted this against alternative explanations for the observed mtDNA pattern, including (i) retained ancestral polymorphism, (ii) relatively recent demographic events producing intraspecific phylogeographic structure, and (iii) long-term reproductive isolation associated with deep phylogenetic divergence.

Our first alternative hypothesis (H1) was that the hares represented more than one distinct evolutionary lineage system (i.e., distinct clusters, haplogroups, phylogroups) undergoing recent divergence with ongoing but clearly reduced nuclear and mt gene flow, i.e., incipient speciation, perhaps in parallel to coat color variation. Our second alternative hypothesis (H2) was that the spatial mt haplotype structure was the result of past introgression from one or more other *Lepus* species, with clearly different mt phylogroups (haplogroups) not necessarily in parallel with the spatial pattern of nuclear gene pool divergence, i.e., recent introgressive hybridization of different species. The presence of phylogenetically divergent mtDNA lineages could also indicate shared ancestral polymorphism due to incomplete lineage sorting in early phases of speciation. Our third alternative hypothesis (H3) was that coat color morphs are rather maintained by local or regional ecological selection and adaptation to habitat characteristics but do not align with reproductively isolated (or partly isolated) evolutionary groups, i.e., species or distinct intraspecific evolutionary units or other markedly partitioned gene pool compartments characterized by very low or interrupted gene flow.

Concordant mt and nuclear gene pool partitioning that aligns with coat color morphs would support (at least some) reproductive isolation and possible species level divergence or incipient speciation. Discordance—deep or spatially structured mtDNA combined with diffuse nuclear genetic admixture—would point to the past introgression of foreign species lineages or retained ancestral polymorphism. Persistent phenotypic variation not paralleled by genetic partitioning would imply the maintenance of morphs by local/regional selection within a single or weakly structured gene pool as expected for regional population differentiation, and intermediate coat color morphs would not show corresponding genetic (nuclear and/or mtDNA) admixture patterns.

## 2. Materials and Methods

### 2.1. Samples

**Figure 1:**
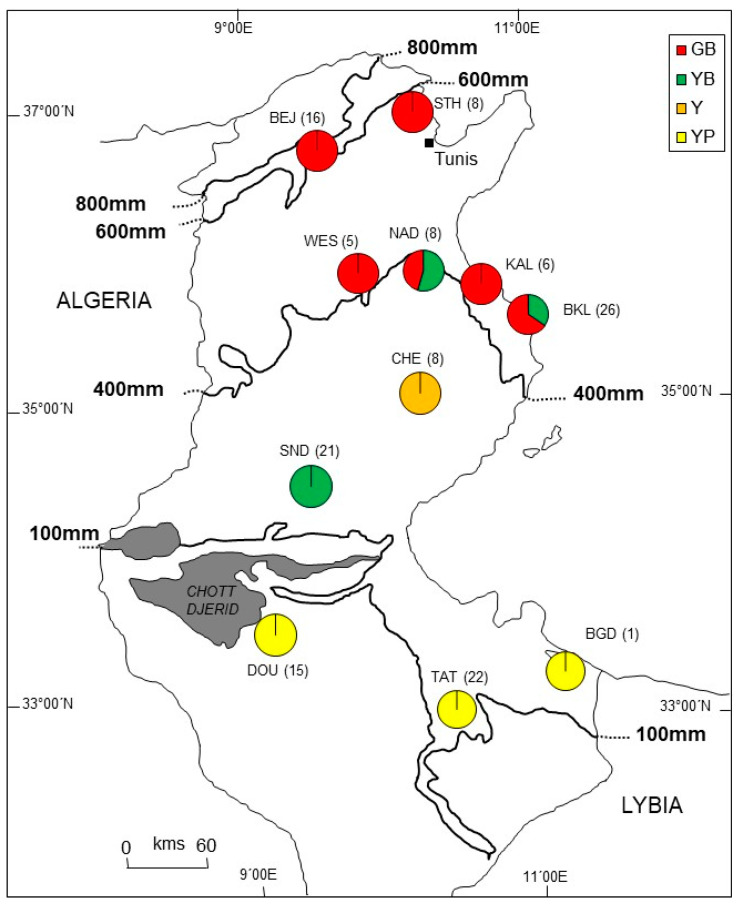
Sampling localities (local populations, sample size in parentheses) of the analyzed Tunisian hares. *STH* Sidi Thabet, *BEJ* Beja, *NAD* Nadhour, *WES* Weslatia, *KAL* Kalâa, *BKL* Bekalta, *CHE* Cherarda, *SND* Sned, *DOU* Douz, *TAT* Tataouine, and *BGD* Ben Guerdène. The pie charts overlaid on the sampling localities indicate the proportion of the four coat color morphs in each local population. GB: grayish brown, YB: yellowish brown, Y: yellowish, and YP: yellowish pale. Mean isohyets for 100 mm, 400 mm, 600 mm, and 800 mm precipitation per year are also given indicating a sharp drop from north to south, i.e., from the northern Mediterranean seaboard to the Sahara. The sedimentary dry basin with the saltwater pan system of Chott el Gharsa, Chott el Djerid, and Chott el Fedjadj in central Tunisia, is indicated in dark gray.

Our study is based on a total of 139 hares, conventionally considered cape hares, *L. capensis* L., 1758, e.g., refs. [5,15,55], collected in 2003–2006 during regular hunts at 10 local sample sites, i.e., “local populations” (Figure 1), in North, Central, and South Tunisia, the three major ecoregions of the country—e.g., refs. [27,56]. Their external phenotypes represented the following four coat color morphs: (1) grayish brown (GB) in northern and central Tunisia; (2) yellowish brown (YB) and (3) yellowish (Y) in central Tunisia; and (4) yellowish pale (YP) in southern Tunisia [53].

Individual tissue samples (liver, skeletal muscles, ear cartilage, tongue) were either stored at −20 °C or in ethanol and DNA was extracted using the GenElute Mammalian Genomic DNA Miniprep kit (Sigma-Aldrich, St. Louis, MO, USA) [53,55,57]. Fourteen microsatellite loci with different levels of polymorphism were screened for this study: Sol08, Sol28, Sol30, Sol33, Sol44, Sat 2, Sat 8 et Sat 12, Lsa 1, Lsa 2, Lsa 3, Lsa 4, Lsa 6, and Lsa 8; for details of PCR amplification and fragment length analysis see [57]. In addition, bidirectional sequencing of a 462 bp-long fragment of the first hypervariable part of the mtDNA control region (mtCR-1) was performed in a total of 116 individuals, as described earlier [53]. The 23 Tunisian cape hare samples from the latter study were included in the present analyses, resulting in total sequences of 139 hares.

### 2.2. Statistical Analysis

#### 2.2.1. Nuclear and Mitochondrial Polymorphism and Differentiation

Prior to the population genetic analyses, we used MICRO-CHECKER v. 2.2.3 [58] to identify possible null alleles, allelic dropout, or scoring failures. We used IDENTIX vers. 1.1., 3 April 2003 [59] to check for possible identical individual composite genotypes potentially indicating identical twins or genotype identity in unrelated individuals by chance; the absence of two identical overall genotypes (in unrelated individuals) would indicate a sufficient degree of molecular resolution of individual discrimination. Allele frequencies, mean number of alleles (A), and observed (H_o_) and expected (H_e_) heterozygosity were calculated for each locus and for each of the 10 local populations with GENETIX 4.05.2, 5 May 2004 [60]; we also used it to calculate genotypic linkage disequilibrium, population-specific estimators of *F_IS_* [61], and respective significance levels for deviation from zero by permutation tests (10,000 permutations), as well as the degrees of differentiation between pairs of populations by Cavalli-Sforza and Edwards (CSE) distances and pairwise *F_ST_* values. We used GenAlEx 6.5 [62] to calculate Jost’s D (Dest) [63], as a supposedly more robust measure of genetic differentiation than *F_ST_*, especially regarding the independence of maximal values from heterozygosity in subpopulations (but see, e.g., ref. [64]). Furthermore, we used GeneClass v. 2.0.h, 2.08.05 ^©^INRA/CIRAD [65] to determine the levels of assignment of individuals to the local population from which they were collected according to their respective composite genotypes—a high level of “correct assignments” would indicate the high suitability of the currently used marker system to describe the underlying (spatial) structure of allelic diversity. We specifically used the Bayesian method of [66] to compute individual likelihood values based on the resampling algorithm of [67] with an assignment threshold of scores of 0.01 and an alpha of 0.01 for 1000 simulated individuals. Also, we used FSTAT [68] to calculate allelic richness (Rs) for each locus and local population, as an estimate of allelic diversity accounting for different sample sizes.

We also performed a 10-factorial correspondence analysis (FCA) as implemented in GENETIX and the resultant individual factor scores were z-transformed and subsequently tested for multivariate variation among the 10 local populations and separately also between the spatial clusters as obtained from our spatial genetic modeling (using GENELAND, see below) in a multivariate general linear model in R [69]. Specifically, we used the following multivariate approach for examining the potential statistical effects of the 10 local populations, the four coat color morphs and the mtCR1 sequences on the combined individual scores of the 10 factors obtained from our FCA: m = lm(cbind(facs1, facs2, facs3, facs4, facs5, facs6, facs7, facs8, facs9, facs10) ~ pop + coat.color + mtCR-1GL, data = dat), where facs 1–10 are the z-transformed individual FCA scores of factors 1–10, pop represents the 10 local populations, and mtCR-1GL represents the two genetic clusters (i.e., populations) obtained from our GENELAND analysis (see below).

For the Tunisian mt-CR1 sequences, DNA polymorphism within local populations (haplotype diversity—h, nucleotide diversity—π, and mean number of pairwise differences—k) was estimated using DNASP version 4.10.8 [70]. In addition, we estimated genetic differentiation among populations using pairwise *F_ST_* values, calculated with Arlequin vers. 3.1. [71] and using randomization analysis to determine statistical significance. We used the same program to calculate the amount of partitioning of relative nuclear and mitochondrial variability among populations by analyses of molecular variance (AMOVAs). Specifically, we ran three AMOVA models to assess the partitioning of genetic variability due to (1) the 10 local populations, (2) the spatial genetic clusters (populations) as obtained from our GENELAND modeling (see below), and (3) the four coat color morphs. The net average distances between populations were calculated by MEGA 6 [72].

#### 2.2.2. Gene Flow Estimation

We used MIGRATE vers. 3.3 [73] to estimate migration rates between pairs of populations in either direction, based on a coalescence theoretical maximum likelihood approach. Specifically, Brownian and sequence models with the following Markov chain settings were used: short chains (short-chains) = 10; trees sampled (short-inc*samples) = 20,000; trees recorded (short-sample) = 200; long chains (long-chains) = 3; trees sampled (long-inc*samples) = 20,000; trees recorded (long-sample) = 200; burn-in per chain = 200,000.

#### 2.2.3. Phylogenetic Analysis for mtCR-1 Sequences

We assessed phylogenetic relationships between the mtCR-1 haplotypes using neighbor-joining (NJ), maximum parsimony (MP), and maximum likelihood (ML) phylogenetic analyses. These analyses included sequences of 5 “*L. schlumbergeri*” from Morocco (access. N° MN175866–MN175870), 4 “*L. saharae*” from Morocco (MN175871–MN175874), 9 *L. c. mediterraneus* from Morocco (MN175857–MN175865), 6 *L. capensis* from northern Egypt west of the Nile delta, 65 *L. saxatilis* from South Africa (AF491386–AF491450), and 41 haplotypes of *L. capensis* from South Africa [74], as well as 2 brown hares, *L. europaeus*, from Anatolia [75], and 2 *L. europaeus* from Germany and Austria [76]. Prior to the MP and NJ analyses, we used Modeltest 3.06 to identify the optimal model of DNA substitution [77], which returned HKY+I+G “HKY85 (TRatio = 4.6713, Shape = 0.2845, Pinvar = 0.5410)” as the best-fit model. The NJ analysis and the MP analysis were performed with the option cited above using PAUP 4.0b10 [78] and used the same program for heuristic ML searches using TBR branch swapping. We estimated ML nodal support by using non-parametric bootstrapping [79], which was restricted to 100 pseudo-replicates because of limited computing time.

In addition, we constructed a median-joining (MJ) network [80] using Network 10.2.0.0 (available at http://www.fluxus-technology.com/sharenet.htm, accessed on 15 June 2025) including all the Tunisian haplotypes; two South African scrub hares, *Lepus saxatilis* (FJ829854, AF491391); two cape hares, *L. c. capensis*, from Darling, Western Cape (DL34 and DL35) [74]; two “*L. mediterraneus*” from Morocco; two “*L. saharae*” from Morocco; and two “*L. schlumbergeri*” [50], as well as two brown hares, *L. europaeus*, from Anatolia [75], and two *L. europaeus* from Germany and Austria [76] as outgroup taxa. All positions were equally weighted, and the parameter ε was set equal to zero. Indels were not considered in the network construction.

In a second analysis, only the Tunisian haplotypes were included, and two sequences of *L. saxatilis* (FJ829854) and *L. capensis* (DL34) [74] were used as outgroups. The best-fit model was also HKY85 (TRatio = 4.6623, Shape = 0.1993, Pinvar = 0). The NJ analysis and the MP analysis were performed with the option cited above using PAUP version 4.0. As a third approach, a maximum likelihood (ML) phylogenetic analysis was performed. We performed heuristic ML searches using TBR branch swapping in PAUP. Finally, we constructed a median-joining (MJ) network [80] using Network 4.2.0.0, including all the Tunisian haplotypes to identify coat color distribution in relation to the evolutionary divergence patterns of mtCR-1 haplotypes.

#### 2.2.4. Isolation by Distance and Association Between Nuclear and Mitochondrial Markers

We evaluated the association between genetic similarity and geographic distance through the regression of *F_ST_*/(*1-F_ST_*) [81] on the logarithm of geographical distances, which is expected to be linear under isolation by distance in two dimensions. The geographical distances were measured as the shortest straight lines between all pairs of local populations, which appears appropriate, given the mostly flat, rolling, or only slightly hilly landscape in our study region. We computed the Mantel correlation coefficient [82], *r*, in GENETIX with 10,000 matrix permutations to determine significances.

#### 2.2.5. Spatial Genetic Models and Genetic Admixture

To identify spatial genetic units (populations) for the nuclear and mitochondrial data sets, we used georeferenced individual genetic data for spatial Bayesian clustering as implemented in GENELAND [83]. We based our spatial models on both markers separately on uncorrelated allele frequencies/haplotypes, with 1 million MCMC iterations, sampling every 100 steps, and numbers of clusters ranging from 1 to 10. All runs were replicated five times. Subsequently, we calculated the same parameters of genetic diversity and differentiation for the obtained populations (i.e., genetic clusters) as for the initial 10 local population samples.

Furthermore, we used STRUCTURE [84] for our microsatellite data to examine the individual genetic admixture and individually based spatial genetic structuring and to determine the most likely number of population groupings (i.e., genetic clusters) compatible with the observed genotypic distribution. The likelihood when assuming different numbers of genetic clusters (K) and the proportion (i.e., percentage—Q) of each K per individual and iteration were calculated under an “admixture model” and “correlated allele frequencies among local populations” with population priors: 500,000 MCMC repetitions after a 100,000 burn-in length, with an initial alpha = 1.0 for K = 1 to 10, and 10 iterations per K. Additionally, we calculated ΔK for each K = 2–9 following Evanno et al. [85].

## 3. Results

### 3.1. Microsatellite Diversity and Population Differentiation

No null alleles or other problematic genotyping was suggested by our Micro-Checker results, conforming with earlier results [54,57,74]. All loci were polymorphic with a total number of 169 alleles and an average of 12.07 alleles per locus. The number of alleles per locus ranged from 6 (Sol33) to 26 (Sol30). Among all detected alleles, 22 (13.02%) were private, i.e., they occurred only in one of the initial 10 local populations, whereas the others were shared between two or more local populations. The observed average heterozygosity ranged from 0.54 (WES) to 0.75 (STH). All pairs of loci were found in genotypic linkage equilibrium. Significant departures of *F_IS_* from zero were registered in six local populations (Table 1). All individual composite genotypes occurred individually, as demonstrated by IDENTIX.

**Figure 2:**
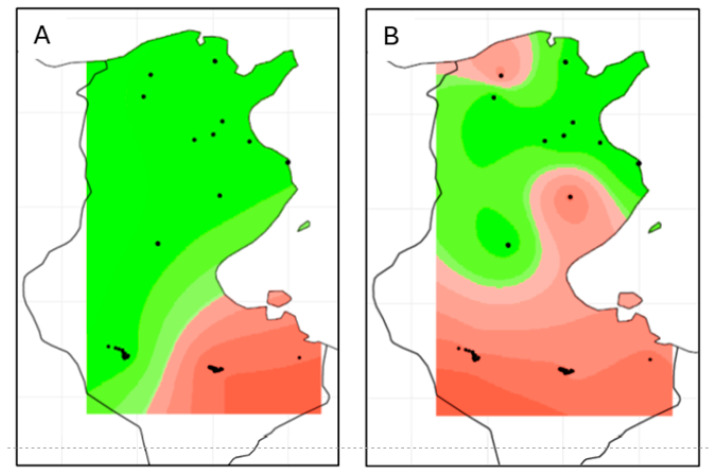
Spatial models of posterior probabilities of genetic cluster membership based on GENELAND analyses of microsatellite loci (**A**) and mtCR1 sequences (**B**). Green—GENELAND population 1; red—GENELAND population 2. Black dots represent sample locations (local populations, see Figure 1).

The spatial clustering for microsatellite genotypes under the uncorrelated model in GENELAND returned as best result K = 2 genetic clusters with high cluster membership probabilities for all individuals; correspondingly, the following two genetic clusters were defined as “GENELAND population 1”, encompassing all individuals from northern, central, and southwestern Tunisia except for population TAT, and “GENELAND population 2”, consisting only of all individuals from the local population TAT in southeastern Tunisia (Figure 2A). The observed heterozygosity for both GENELAND clusters was 0.63 and population specific allelic richness (Rs) amounted to 8.784 for GENELAND population 1 and to 8.506 for GENELAND population 2. Notably, both clusters showed significant *F_IS_* departure from zero (Table 1).

Pairwise *F*_ST_ values of relative genetic differentiation between all local populations ranged from 0.007 (KAL-CHE) to 0.074 (SND-WES) (Table 2) with 19 (42.22%) values significantly above zero, when accounting for multiple testing by strict Bonferroni corrections (e.g., ref. [86]). Overall relative genetic differentiation was small but significant (*F_ST_* = 0.0377, *p* < 0.0001). The CSE values ranged from 0.057 (DOU-TAT) to 0.12 (CHE-WES) (Table 2) with 15 (33.33%) pairwise comparisons being significantly above zero under strict Bonferroni corrections. Differentiation of the local populations as determined by mean *Dest* values (Table 2) across the 14 loci was similarly relatively low, ranging between 0.019 (KAL-CHE) and 0.257 (BKL-TAT). For the two GENELAND populations (genetic clusters), pairwise *F_ST_*, CSE, and *Dest* values were 0.0435, 0.045, and 0.166, respectively. All three tests returned significant differences between the two GENELAND populations (clusters).

All but two (i.e., 98.56% *n* = 139) individual composite microsatellite genotypes were assigned to the local population where they were collected by our GeneClass runs. However, two composite genotypes of NAD individuals were assigned to Douz/Kebili and Kalaa, with probabilities 6.99% and 6.42% higher than for NAD, respectively.

As regards geographic variability, our AMOVA models showed that allelic variation occurred mainly within local populations (Table 3). The results of the first AMOVA model, in which populations were categorized according to their original local sampling areas; showed a low contribution (4.73%) to the total genetic variation. Similarly, the second AMOVA model indicated no (2.29%, not significant) partitioning between the two GENELAND populations and a low level of variance partitioning between the local populations within the two GENELAND populations (3.99%; *p* < 0.001); rather, a high (93.72%, *p* < 0.0001) level of partitioning of allelic variation was found between individuals within the two GENELAND clusters. Our third AMOVA model indicated no significant partitioning of the total allelic variability encountered presently among the four coat color types (Table 3).

Estimates of migrating individuals among the 10 local populations in our MIGRATE simulations yielded for the microsatellite data bidirectional pairwise *Nm* values between 0.148 and 2.376 individuals per generation with 37.78% of the values above 1.0 (see Table 4); the Nm value of migration from spatial GENELAND populations 1 to 2 amounted to 4.389 and to 1.468 in the opposite direction. Our Mantel test showed that the patterns of nuclear gene pool divergence did not follow an isolation by distance model (r = 0.178, *p* = 0.165). According to the FCA, only 23.64% of the currently observed overall allelic variation was conveyed by the first 10 factors (factor 1 = 2.99%, factor 2 = 2.67%, factor 3 = 2.52%, factor 4 = 2.43%, factor 5 = 2.38%, factor 6 = 2. 24%, factor 7 = 2.18%, factor 8 = 2.11%, factor 9 = 2.08%, factor 10 = 2.04%).

**Figure 3:**
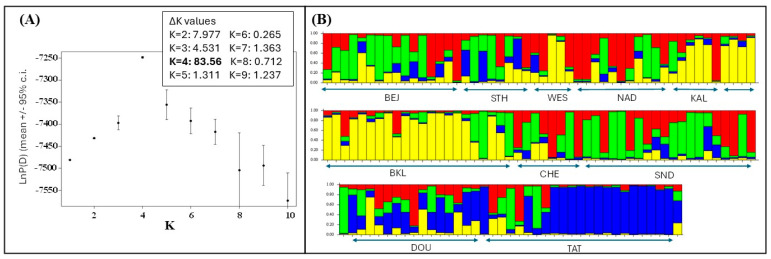
STRUCTURE results—(**A**) Likelihood (LnP(D)) distribution of K (number of genetic clusters underlying the overall data set), for K ranging from 1 to 10; means and 95% confidence intervals are given; ΔK values [85] for each K from 2 to 9 are also given—the highest (peak) ΔK value (K = 4) is indicative of the most likely number of clusters; (**B**) bar charts of individual genetic grouping and admixture when assuming K = 4 for correlated allele frequencies and admixture models without population priors. Local population acronyms are given according to Figure 1.

The multivariate general linear model (MANOVA) of the individual FCA scores in the range of 1–10 (Table 5) indicated a significant effect by local populations but not by coat colors or individual mtCR1-haplotype populations as obtained by our GENELAND modeling. Our STRUCTURE analysis indicated that the overall genotype dataset was best partitioned into four genetic clusters (K = 4), when based on the L/nPD values and the associated variance. However, neither the individual coat color type nor the origin of individuals from the 10 local populations were reflected by the four genetic clusters identified (Figure 3).

### 3.2. mtCR-1 Diversity, Differentiation, and Phylogeny

The 462 bp fragment of mtCR-1 showed 78 variable sites (16.88%), 58 (12.55%) of which were parsimony informative, and 3 of which were indels (43, 44, and 45). The sequences yielded a total of 60 haplotypes (Table 6) in the overall Tunisian population with 34 (57%) private ones (i.e., occurring in a single local population sample). Haplotype diversity was high (h = 0.966 ± 0.007, standard error) but nucleotide diversity (π = 0.0273 ± 0.0009) was relatively small. When calculated for single local populations, diversity index values ranged between h = 0.709—0.981, π = 0.00531—0.02820, and k = 2.441—12.942 (Table 6).

**Figure 4:**
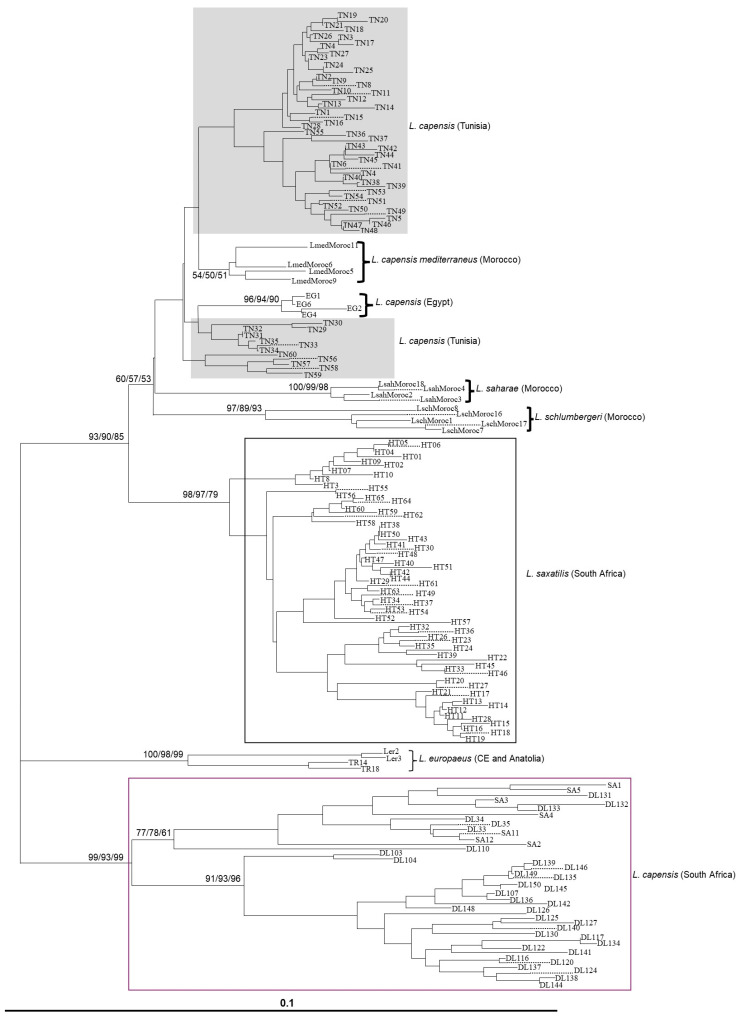
Neighbor-joining dendrogram based on HKY85 distances including several hare sequences with the Tunisian mitochondrial sequences (Materials and Methods Section). Percentage of bootstrap values are given only for major clades for NJ and MP analyses, if above 50%.

**Figure 5:**
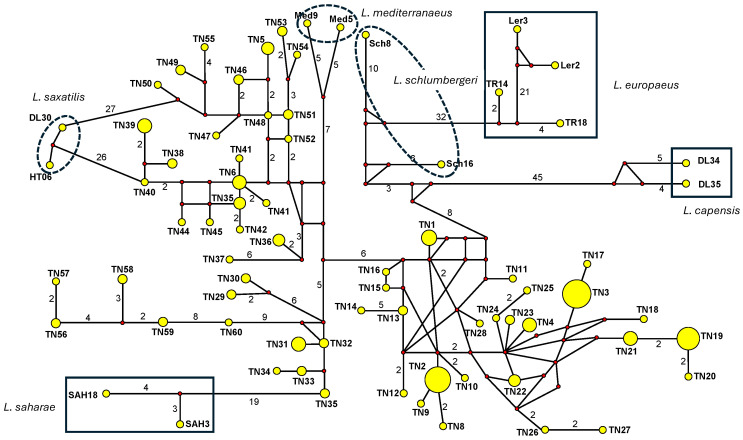
Median-joining network showing the relationships among Tunisian haplotypes and several hare species (see Materials and Methods Section). Relative haplotype frequencies correspond to haplotype circle size (see Table 1). Numbers on lines connecting haplotypes indicate numbers of mutation changes. Small open circle indicates inferred haplotype.

The overall phylogenetic analyses of the Tunisian mt-CR1 haplotypes, together with those from GenBank, showed that all Tunisian haplotypes clustered with *L. capensis mediterraneus* from Morocco and the Egyptian sequences (Figure 4). The closest sequences to this phylogroup were those of “*L. saharae*” and “*L. schlumbergeri*” from Morocco. The next closest to this North African phylogroup were sequences of the South African scrub hare, *L. saxatlis* rather than South African *L. capensis*. The close relationship of the North African haplotypes and *L. saxatilis* was confirmed by the median-joining network (Figure 5).

Our GENELAND approach also identified two clusters for mtCR-1 sequences. The first cluster, i.e., “mt GENELAND cluster 1”, was composed of 77 individuals mainly from the NT and CT regions, and “mt GENELAND cluster 2” was composed of 61 individuals with 35 out of 38 individuals (92.11%) from the DOU and TAT populations in South Tunisia.

Pairwise *F_ST_* values between the 10 local populations ranged from −0.04463 (STH-NAD) to 0.70934 (BKL-CHE), with 14 out of 45 comparisons (31.11%) differing significantly from zero after Bonferroni correction (Table 2). For the two GENELAND clusters *F_ST_* amounted to 0.0508 (*p* < 0.001). The net average distance between the 10 local populations ranged from −0.0003 ± 0.0006 (WES-NAD) to 0.0223 ± 0.006 (BEJ-STH) and was 0.015 ± 0.0038 between the two mt GENELAND clusters.

Our AMOVA models showed that more variation occurred among populations for the Tunisian mt-CR1 sequences compared to microsatellite loci (Table 3). The first AMOVA revealed that 18.44% (*p* < 0.05) of the total variance was distributed among the 10 local populations. The second AMOVA model indicated that 41.28% (*p* < 0.01) of the total variance was recorded between the two mt GENELAND clusters (Table 3), and according to our third model there was no significant partitioning (−2.19%) among individuals with different coat color morphs (Table 3).

Estimates of gene flow among the 10 local populations were generally low, according to the modeled numbers of migrating individuals per generation based on our mtCR-1 sequences, with no migrating individuals (i.e., Nm = 0.000) in 80% of all comparisons. However, the number of migrants per generation was 19.24 between mt GENELAND cluster 1 and mt GENELAND cluster 2 and 46.44 in the opposite direction.

**Figure 6:**
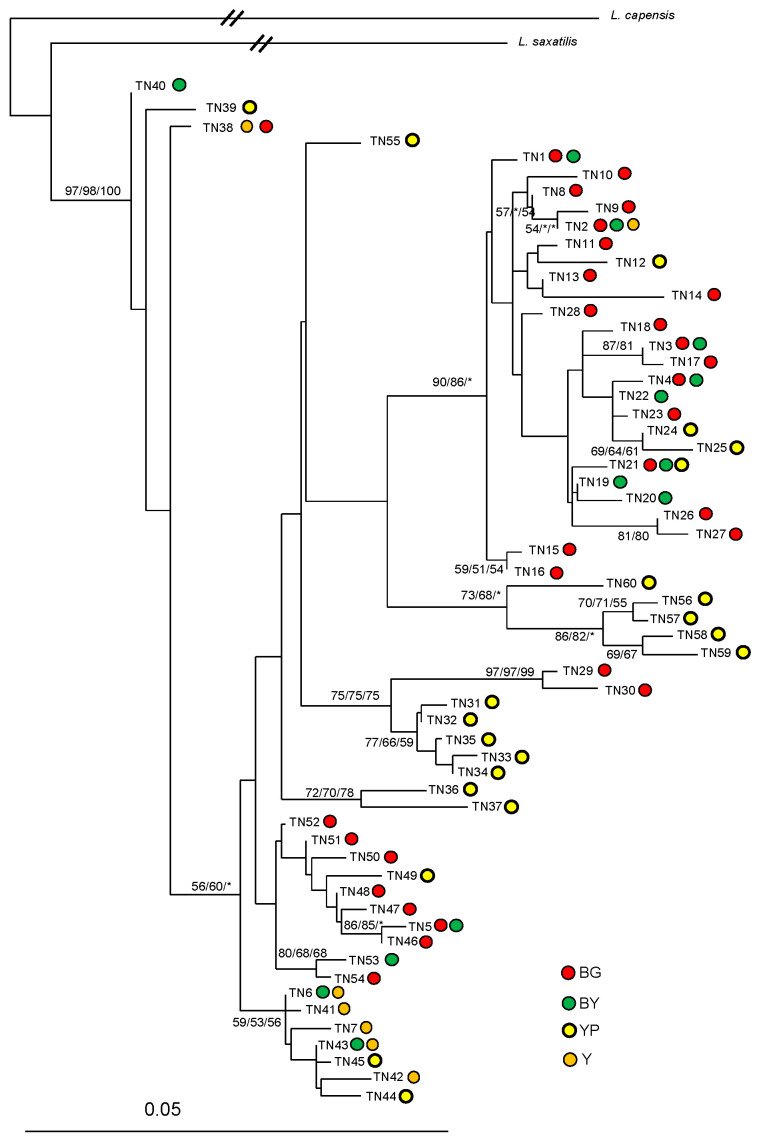
Neighbor-joining dendrogram based on HKY85 of the Tunisian haplotypes and associated coat color phenotype, YP—yellowish pale, Y—yellow, GB—grayish brown, and YB—yellowish brown. Percentage of bootstrap values are given for NJ/MP/ML, if >50.

**Figure 7:**
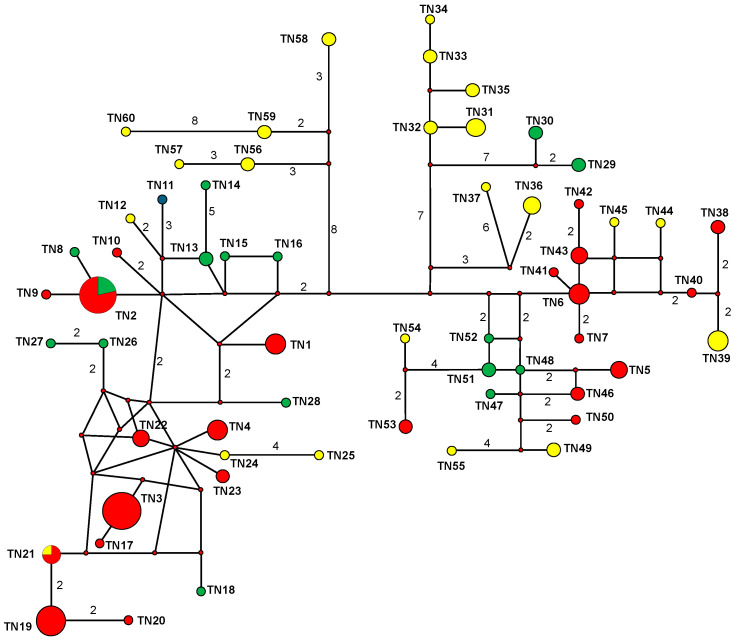
Median-joining network as model of evolutionary relationships among Tunisian haplotypes and their distribution across the three major climatic regions in Tunisia. Relative haplotype frequencies correspond to haplotype circle size. Numbers on lines connecting haplotypes indicate numbers of mutation changes. Small open circle indicates inferred haplotype. Green: Mediterranean North Tunisia; red: semi-arid Central Tunisia; yellow: arid South Tunisia.

Our NJ, ML, and BI analyses of the Tunisian haplotypes (Figure 6) showed a shallow but significant separation of the encountered lineages into up to six haplogroups with relatively high bootstrap support. However, there was no clear correspondence between the haplotype distribution and the coat color morphs of individuals or their geographical origin, i.e., local population (Figure 6 and Figure 7). In contrast, the NJ network recovered the two mt GENELAND clusters (Figure 7).

We found no isolation by distance for the Tunisian sequences (r = 0.129, *p* = 0.313), and the patterns of geographical differentiation, indicated by pairwise *F_ST_* values among the 10 local populations obtained for the microsatellites and the mitochondrial sequences, were not significantly correlated (r = −0.091, *p* = 0.6580, Mantel test).

## 4. Discussion

None of our results, for either the nuclear or the mitochondrial (mt) gene pool, met the expectations of our alternative hypotheses H1 and H2, i.e., we did not find any evidence of incipient speciation, ancient introgression by foreign lineages characteristic of other species, shared ancestral mt polymorphism, or presence of more than one hare species. Rather, all findings conform with expectations of our null hypothesis (H0), i.e., no significant genetic differentiation into more than one species. The currently observed level of nuclear and mt gene flow in the hares from Tunisia corresponds to the level usually observed among local or regional populations of a single hare (*Lepus*) species that has largely evolved under “genetically undisturbed conditions” and that exhibits spatial variation and differentiation without signals of strong drift effects and a regular—i.e., non-reticular—phylogenetic history and without or with larger phylogenetic gaps—compare e.g., refs. [87,88,89,90,91] (for *L. capensis*, *L. europaeus*, *L. timidus*). Furthermore, we found no parallels among the four different coat color morphs observed in the Tunisian hares and the general pattern of their genetic differentiation. This conformed to preliminary findings [53] and corresponds to our hypothesis (H3) that the four coat color morphs largely reflect adaptation to environmental (habitat types, climate) conditions, supposedly through a positive selection of genes that are not representative of larger compartments of the entire gene pool.

### 4.1. Coat Colors and Genetic Differentiation

In hares, the variation in coat color and patterns may be based on comparatively few genes, as e.g., in dogs [92], and changes can occur remarkably quickly; for instance, Fennoscandian mountain hares, *L. timidus*, have changed their fur characteristics within less than 100 generations after their introduction to the Atlantic Faroe Islands, by introgression, positive selection, and selective sweeps in regulatory genes [93] (see also [94] for *L. americanus*). Intraspecific seasonal variation in coat colors is also known from mountain hares, *L. timidus*, which generally change their brownish, grayish, blueish hue of summer coats into white winter coats, except for Irish specimens, *L. t. hibernicus*, which maintain their brown coat over winter, likely due to adaptation to the regional habitat with little snow cover. Their genetic differentiation from other mountain hare subspecies is of little magnitude, compared to the large molecular variation within the whole species, e.g., refs. [88,90]. In Japanese hares, *L. brachyurus*, the two winter coat color morphotypes are not related to population genetic divergence [95].

Our finding that overall genetic differentiation does not parallel coat color variation in Tunisian hares aligns with the results from brown hares, *L. europaeus*, in Anatolia [79] and hares from other parts of the Near East [46,96,97,98], where no pronounced gene pool divergence was detected among different coat color morphs. The same applies to Mexican populations of *L. californicus* [99]. Notably, hares from along the eastern Mediterranean seaboard are considered a single species despite their markedly different external phenotypes (including different coat colors), but to our understanding the species is still not clear—brown hare, *L. europaeus*, or cape hare, *L. capensis* [46,96,97,98,99]. Microsatellite-based population genetic data revealed high gene flow and a close genetic relationship between hares from northern and southern regions [57]. Northern populations exhibited phenotypes typical of *L. europaeus*, while southern populations from the desert areas (Arava Valley) showed phenotypes characteristic of *L. capensis*. In the central–southern semi-arid regions, intermediate phenotypes were observed, accompanied by corresponding variation in body size consistent with Bergmann’s rule [97,98]. Their close genetic relationship, regardless of their external phenotype, was also confirmed by epigenetic dental characters, which, however, could not clearly assign the hares to either cape hares or brown hares [100]. Anatolian brown hares with a yellowish coat color, specifically from the Şanlıurfa region in southern Anatolia, feature mt D-loop sequences that form a small phylogroup nested within the network of typical Anatolian lineages. However, given the high level of genetic differentiation among all Anatolian sequences, the phylogenetic separation of that latter mt phylogroup representing hares with yellowish coat color is negligible [75]. Also, nuclear gene pool differentiation between hares with brownish and yellowish coats in Anatolia is at the level of local or regional conspecific populations and indicates substantial gene flow—e.g., ref. [101].

In principle, a similarly low level of nuclear gene pool differentiation—characteristic of regional or local populations—has been observed among several subspecies of cape hares, *L. capensis*, in South Africa, despite their variation in coat color, patterning, and other morphological traits [74,87,102,103,104] (and the literature cited therein). A quite substantial phylogenetic gap in mtCR1-haplotypes was not paralleled by microsatellite-based gene pool divergence; rather, relatively high gene flow was characteristic for all cape hares studied in South Africa [74] (see e.g. also [75,101,105] and the literature cited therein for high gene flow despite a phylogenetic mtDNA gap in *L. europaeus*).

In Italian hares, L. *corsicanus*, which are genetically distinct from *L. europaeus*, and that only rarely show signals of introgression by the latter species [106], coat color shows little variation [107], while *L. granatensis* from the Iberian Peninsula seem to vary somewhat more in their coat color with darker hues in northern populations [108]. To our knowledge, a detailed study of the genetic basis of that variation is not available, specifically if the late Pleistocene introgresson of *L. timidus* into northern populations exerted an effect on the variable hue [109,110]. Introgression may suggest darker coat colors in introgressed populations, as, e.g., in hares (*L. europaeus*, *L. capensis*, *L. tolai*) from the Near/Middle East or in our currently studied Tunisian hares. It may also indicate adaptive camouflage (e.g., hiding from predators or birds of prey) or/and adaptation to thermoregulatory conditions (exposure to sun or/and ambient temperature), with darker hues being more advantageous in wetter and colder environments—e.g., ref. [111]. Very dark/blackish—melanistic—forms in temperate/cold (as in “*L. melainus”)* and warm (as in “*L. insularis”*) environments have earlier been considered separate species but are now considered only subspecies according to genetic studies [112,113,114,115].

The currently observed coat color variants in the Tunisian hares may result from both thermoregulatory adaptation to local climatic conditions and adaptation to reduce the predation pressure due to better camouflage. However, our genetic data show that they have no indicative taxonomic value, not even on the intraspecific level, as we did not find any indications of recent genetic diversification in the sense of incipient speciation concordant with coat color morphs, nor signals of ancestral introgression or hybridization of different species in our study area that may help to explain coat color variation (for more detailed arguments see below).

### 4.2. Gene Flow, Spatial Genetics, and Systematic Implications

Gene flow and genetic differentiation among the hares in our study were in the lower range typically found for inter-population comparisons in various *Lepus* species with similar sets of markers, e.g., refs. [88,101,116], when based both on our initial local population approach and our GENELAND models. Some population genetic data—e.g., for brown hares—clearly indicated higher levels of relative genetic differentiation, especially for populations that most likely have experienced founder effects due to anthropogenic introductions or natural immigrations by pioneer populations [117].

Our results of genetic structure and admixture indicated variable levels of individual genetic admixture but only a tendency of changes in the admixture patterns across our study area rather than a sharp difference among local populations—again in line with the overall low level of genetic differentiation. Nevertheless, and somewhat unexpectedly, nearly half of the individuals showed very little genetic admixture, and within single local populations it was not uncommon to find little-admixed individuals assigned to different genetic clusters. However, within local populations, no clear correspondence between STRUCTURE cluster assignment and coat color was observed. For example, in TET and DOU only a single coat color (YP, yellow pale) occurred, yet both genetically almost pure and admixed hares were present. And in the local populations BEJ, STH, WES, and KAL all individuals with different genetic admixture patterns showed a grayish-brown coat color (GB). Nevertheless, it is noteworthy that 60% of all local populations and the two GENELAND genetic clusters (populations) showed significant inbreeding coefficients, *Fis*. This points towards even more local genetic structuring (i.e., a Wahlund effect) than expected by our initial spatial sample regime, but it further underlines the effectiveness of our marker system to trace spatial genetic differentiation. However, because only five of the 10 local populations were represented by ≥15 individuals, the smaller sample sizes in the remaining populations should be taken into consideration when evaluating estimates of genetic diversity, F-statistics, clustering patterns, and gene flow parameters.

In accordance with the above interpretation of our admixture results, both our AMOVA models and our individual FCA scores MANOVA models concordantly demonstrated that the four coat color morphs of the Tunisian hares did not have a significant effect on the nuclear gene pool composition as reflected by our microsatellite data. The MANOVA models also indicated no effect of the two mtCR1 populations as obtained from our GENELAND models on the individual FCA scores, which, however, reflected a relatively small partition of the total allelic variance found in the present data. No relationship between nuclear and mt gene pools was also found by our Mantel test. This underlines the problematic use of only mtDNA and phenotypical/morphological characteristics for systematic conclusions within the genus *Lepus*. Morphological traits such as skull size and shape can evolve rapidly within a single species under certain environmental conditions, sometimes in fewer than 20 generations—at least slightly. This was found in a preliminary study of brown hares, *L. europaeus*, from a breeding station [118] without inbreeding or other substantial changes in their population genetic make-up [119] but under “pre-domestication conditions”. Therefore, we are skeptical when the description of new *Lepus* species from Northwest Africa, such as “*L. saharae*”, “*L. mediterraneus*”, “*L. schlumbergeri*” [50], and the late Pleistocene “*L. berbericus*” [51], is based only on body or skull size and minor phenotypic characteristics such as minor dental characters or fur color and pattern in addition to mtDNA characteristics. Until convincing nuclear gene pool data are available to demonstrate their distinct genetic separation from other African species/forms—specifically *L. capensis* and *L. victoriae* or *L. saxatilis*—we prefer to provisionally retain them within *L. capensis*. We maintain this provisional systematic position despite their closer mtDNA-based phylogenetic relationship with *L. saxatilis*/*victoriae*, in order to reduce taxonomic confusion by repeated renaming should future genetic or genomic evidence support alternative species assignments (see below and [53]).

The significant effect of local populations on the coat color morphs, when accounting for microphylogenetic, i.e., mtCR1 genetic populations and current population genetic differentiation patterns, is not incongruent with the interpretation of positive selection of certain alleles/genotypes of specific coat color genes and their adaptation to local environments [111] (e.g. [95] for Japanese *L. brachyurus*). Positive selection of other phenotypic characteristics, like minor skull and dental traits, or genetic drift effects may regionally also result in rapid phenotypic changes, such as in *L. insularis* that is now considered a subspecies of *L. californicus* [112] or the late Pleistocene “*L. tanaiticus”* that may be considered a subspecies of *L. timidus* [120]. Such minor phenotypic changes may, however, not be paralleled by major gene pool changes and by concomitant reproductive barriers between regional forms and therefore not justify species rank (under a biological and/or genetic species concept).

### 4.3. mt DNA Haplotypes, Phylogeny, and Phylogeography

Higher levels of divergence in mtDNA than between nuclear gene pools are frequently reported for various hare species, independently of whether foreign introgressed lineages or ancestral polymorphism was found, e.g., refs. [57,87,121,122] (for *L. europaeus*, *L. timidus*, *L. capensis*); it is commonly interpreted as resulting from higher female philopatry. Such philopatry-based scenarios of stronger spatial partitioning of mtDNA may be reinforced and accumulate by the above cited “Mother’s Curse effect” over generations and lead to more distinct genetic/phylogenetic differentiation signals by mtDNA markers compared to nuclear markers and may bias systematic conclusions towards “oversplitting”.

The numerous mtCR1 haplotypes identified in the current study exhibit only shallow differentiation between two phylogroups, as already suggested [53], representing “category IV” in the terminology of Avise [123], i.e., a shallow gene tree, with largely sympatric lineages. The haplotypes of these two phylogroups are not geographically concordant and do not align with the two genetic populations identified by our GENELAND models. Rather, haplotypes of different phylogenetic positions are distributed across the whole study region, concordant with the high gene flow found in our coalescence theory-based model. The marginal evolutionary divergence observed between the two phylogroups corresponds closely with the southern–central Tunisian salt-pan complex (Chott el Gharsa, Chott el Djerid, and Chott el Fedjadj), a region whose severe environmental conditions have historically restricted the presence of hares. This geographic setting may have kept the two currently observed phylogenetic populations separated during the late Pleistocene and/or early Holocene and our data are not incongruent with the hypothesis that they have come into contact in our study region only very recently in evolutionary times and have merged to a very large extent.

Our phylogenetic mtCR1 lineage models revealed neither any “foreign lineages” indicative of shared ancestral polymorphism due to incomplete lineage sorting nor any consistent spatial divergence pattern matching our GENELAND models or corresponding to the coat color morphs that would otherwise indicate incipient speciation. Rather, somewhat surprisingly in line with our earlier findings [53], the closest mtCR1 haplotypes are those of *L. saxatilis*, from (the Western Cape in) South Africa, apart from the other North African haplotypes that have recently been considered separate species (“*L. saharae*”, *L. schlumbergeri*”, *L. mediterraneus*”, and the haplotypes of Egyptian hares from west of the Nile Delta). All haplotypes of the latter North African forms can be easily understood as merely representing other regional evolutionary clusters of a complex evolutionary network encompassing large areas of North Africa and representing only one species, i.e., either *L. capensis* or *L. victoriae*, or *L. saxatilis*, pending further phylogenetic and phylogeographic data of both nuclear and mt gene pools. This hypothesis is supported by the marked evolutionary divergence among Tunisian mtCR1 haplotypes from our study area, coupled with their relatively limited phylogenetic differentiation from *L. saharae*, *L. schlumbergeri*, and *L. mediterraneus*. Specifically, the closest haplotypes differ by 22 substitutions (*L. saharae*: SAH3–TN35), 17 (*L. schlumbergeri*: Sch16–TN1), and 16 (*L. mediterraneus*: Med5 or 9–TN6), whereas the maximum divergence observed within Tunisian haplotypes themselves is 19 substitutions (TN20–TN57), after accounting for multiple hits.

Notably, there is no major phylogenetic gap between the TN20 and TN57 haplotypes. It is conceivable that the complex evolutionary network of mt lineages revealed by our NETWORK model is not confined to Tunisia but extends across broader regions of North Africa; data from Algeria and neighboring areas may phylogenetically integrate the haplotypes “*L. saharae*,” “*L. schlumbergeri*,” and “*L. mediterraneus*” into a unified and intricate evolutionary mt lineage system possibly without phylogenetic discontinuities. This is at least already suggested by our tree-based phylogenetic results for “*L. mediterraneus*”. Whether the Tunisian haplotypes are indicative of “*L. mediterraneus*” or *L. capensis mediterraneus*, or another species (i.e., *L. victoriae*/*L. saxatilis*) needs to be investigated by nuclear gene pool analyses or genomic analyses. A large and reticulated mtCR1 haplotype network—with numerous substitutions separating the most divergent haplotypes—was also observed in brown hares (*Lepus europaeus*) from Anatolia and Europe. Despite this diversity, the phylogenetic separation between Anatolian and European haplotypes is small (e.g., [105]), and nuclear markers indicate high gene flow both within each region and between the regions.

Hence, for North African hares, a similarly large intraspecific evolutionary network of divergent mt lineages may be expected as for brown hares and for South African cape hares, *L. capensis*, which were all from populations with high levels of gene flow in the nuclear gene pools [74] across the whole of South Africa, despite distinct differences in their external phenotypes [102]. The level of mtCR1 lineage divergence of the latter cape hares is even larger than the evolutionary mtDNA divergence observed for all currently studied North African lineages and those of brown hare, *L. europaeus*, haplotypes. Moreover, given the closer phylogenetic relationship between the North African mt linages and *L. saxatilis* lineages of South African origin rather than *L. capensis* lineages, an ancestral allochthonous regional mt capture of the former lineages may not be ruled out. Support for this hypothesis may come from the close mitochondrial phylogenetic relationships between *L. saxatilis* and *L. europaeus*, as well as their shared affinities with the three Ethiopian hare species—*L. habessinicus*, *L. fagani*, and *L. starcki* [54,124]. These relationships are consistent with the environmental conditions of the early Holocene “Green Sahara,” which for thousands of generations provided extensive migration corridors for open-grassland and dry-savanna mammals, enabling gene flow between North Africa and large parts of what is now sub-Saharan Africa [125,126,127].

## 5. Conclusions

Our combined population–genetic and phylogenetic analyses show that Tunisian hares exhibit high gene flow, weak nuclear differentiation, and only shallow mitochondrial divergence, despite their marked coat color variation. Microsatellite data revealed limited spatial genetic structure and widespread admixture, with no correspondence between genetic clusters and external phenotypes. The two mtCR1 phylogroups identified are only marginally differentiated, consistent with recent secondary contact and extensive historical gene flow. In addition, neither nuclear nor mitochondrial markers provide evidence for incipient speciation or for the presence of distinct evolutionary lineages within Tunisia. The mtDNA patterns instead reflect a broader North African network of divergent but interconnected lineages, like patterns known from other *Lepus* species with reticulate evolutionary histories.

Given these results, species delimitation in North African hares cannot rely on phenotypes and mtDNA alone. Under a biological and/or genetic species concept, a robust assessment of species boundaries in this region requires comprehensive phylogeographic sampling and nuclear genomic data, ideally across all African taxa currently assigned to *L. capensis*, *L. saxatilis*, and *L. victoriae*, and including comparisons with East African species. Our study underscores the need for integrative approaches when dealing with taxa characterized by high phenotypic plasticity, incomplete lineage sorting, and recurrent hybridization—processes that are common in the genus *Lepus*.

## Figures and Tables

**Table 1:** Genetic diversity of Tunisian hare populations based on microsatellite loci.

Populations	A	Rs	*F_IS_*	He	Ho
BEJ (16)	7.86	4.830	0.164 *	0.713	0.619
STH (08)	5.68	4.935	0.055	0.741	0.750
WES (05)	4.36	4.357	0.219 *	0.610	0.543
NAD (08)	6.71	4.781	0.098	0.710	0.666
KAL (06)	4.64	4.350	0.125	0.675	0.643
BKL (26)	7.79	4.717	0.139 *	0.710	0.625
CHE (08)	5.79	4.758	0.116	0.701	0.667
SND (21)	7.29	4.759	0.149 *	0.740	0.648
DOU (15)	7.79	5.225	0.241 *	0.760	0.602
TAT (22)	8.57	5.127	0.220 *	0.779	0.626
GL Cluster 1 (116)	11.5	8.784	0.175 ***	0.771	0.639
GL Cluster 2 (22)	8.64	8.506	0.207 ***	0.780	0.636

A, mean number of alleles per locus; Rs, allelic richness; H_e_, expected heterozygosity, corrected for small samples; H_o,_ observed heterozygosity; *F_IS_*, inbreeding coefficient. Values that showed significant departure from zero for the *F_IS_* are indicated with * and *** (α < 0.05, α < 0.005 after strict Bonferroni correction, respectively). GL populations 1 and 2 are the two genetic populations (clusters) as obtained by GENELAND.

**Table 2:** Genetic differentiation among hare populations.

	BEJ	STH	WES	NAD	KAL	BKL	CHE	SND	DOU	TAT
BEJ	-	0.025 0.083 0.082 *	0.034 0.093 0.093 *	0.016 0.048 0.059 *	0.034 0.099 0.078	0.049 * 0.140 * 0.056	0.019 0.058 0.072 *	0.031 * 0.096 * 0.059	0.038 * 0.130 * 0.074	0.059 * 0.214 * 0.071
STH	0.035 0.022	-	0.047 0.146 0.116 *	0.037 0.129 0.093 *	0.044 0.149 0.112	0.044 * 0.140 * 0.088	0.018 0.064 0.097 *	0.022 0.081 0.083	0.018 0.074 0.093 *	0.028 0.120 0.089 *
WES	−0.015 0.011	0.025 0.000	-	0.057 0.157 0.106 *	0.036 0.093 0.114	0.018 0.044 0.085 *	0.066 * 0.188 * 0.120 *	0.074 * 0.223 * 0.117	0.027 0.091 0.100 *	0.071 * 0.249 * 0.114 *
NAD	0.078 0.016	−0.045 0.002	0.021 −0.000	-	0.025 0.074 0.084	0.051 * 0.151 * 0.070	0.019 0.059 0.078 *	0.022 0.069 0.059 *	0.029 0.103 0.072 *	0.067 * 0.254 * 0.087
KAL	0.072 0.020	−0.008 0.001	0.076 −0.001	−0.002 0.001	-	0.036 0.099 * 0.074	0.007 0.019 0.069	0.046 * 0.147 * 0.093	0.032 0.118 * 0.093	0.060 * 0.227 * 0.094
BKL	0.305 * 0.023	0.350 * 0.004	0.308 0.003	0.374 * 0.004	0.255 * 0.002	-	0.064 * 0.193 * 0.084	0.052 * 0.157 * 0.069	0.031 * 0.097 * 0.069	0.074 * 0.257 * 0.073
CHE	0.260 * 0.019	0.423 * 0.001	0.267 −0.001	0.380 0.001	0.503 0.000	0.710 * 0.003	-	0.021 0.067 0.072 *	0.027 0.098 0.076 *	0.059 * 0.230 * 0.082 *
SND	0.120 0.008	0.201 0.005	0.052 −0.001	0.222 0.001	0.173 0.003	0.280 * 0.005	0.491 * 0.002	-	0.018 0.062 0.062	0.056 * 0.214 * 0.071
DOU	0.161 0.001	0.268 * 0.022	0.166 0.010	0.294 * 0.015	0.326 * 0.019	0.556 * 0.021	0.144 * 0.018	0.332 * 0.006	-	0.027 * 0.110 0.057 *
TAT	0.081 * 0.003	0.141 * 0.017	0.076 0.008	0.175 * 0.012	0.202 * 0.014	0.411 * 0.017	0.228 * 0.014	0.233 * 0.005	0.070 0.004	-

Upper diagonal: pairwise *F_ST_* values according to Weir and Cockerham [61] (first value), pairwise Dest values [63] (second value) and pairwise CSE values (third value) as obtained from microsatellite data. Lower diagonal: pairwise *F_ST_* values (first value) and net between population mean distances (second value) as calculated from mtCR-1 sequences (see Materials and Methods Section). *—Significantly above zero at *p* < 0.05, after strict Bonferroni correction.

**Table 3:** Hierarchical AMOVAs revealing partitioning of allelic and haplotypic variation at microsatellite loci and mtCR-1 sequences.

	Source of Variation	Microsatellite Loci		mtCR-1 Sequences	
		df	% of Variation	df	% of Variation
local populations	among local populations	9	4.73 *	9	18.44 *
	within local populations	266	95.27 *	128	81.56 *
GENLAND populations (genetic clusters)	between GENELAND populations (clusters)	1	2.29	1	41.28 **
	among local populations within clusters	8	3.99 ***	10	6.72 ***
	within local populations	268	93.72 ***	125	52.00 ***

Significance levels: * p < 0.05, ** p < 0.01, and *** *p* < 0.001.

**Table 4:** Numbers of migrating individuals as calculated by the MIGRATE program for microsatellite loci (upper value) and mtCR-1 sequences (lower value) in either direction. The source populations of migration are indicated in the first column and respective receiving populations are indicated in the upper line with the population acronyms.

	BEJ	STH	WES	NAD	KAL	BKL	CHE	SND	DOU	TAT
BEJ	-	0.7894 0.0000	1.0049 11.7435	1.2486 0.0000	0.9330 0.0000	1.2918 0.0000	0.7321 0.0000	1.8659 0.0000	0.6746 76.3352	1.0624 0.0000
STH	0.7438 0.0000	-	0.5722 0.0000	1.0298 36.4992	0.4863 0.0000	0.9011 0.0000	0.7295 0.0000	0.7579 0.0000	0.8010 0.0000	0.6576 0.0000
WES	0.2574 209.0571	1.1275 0.0000	-	2.2751 139.3701	0.3165 0.0000	1.1078 174.2079	0.1780 0.0000	0.2375 0.0000	0.4947 0.0000	1.0683 0.0000
NAD	1.4850 0.0000	0.3559 535.0841	0.6382 0.0000	-	0.9204 0.0000	0.3559 137.3483	0.8221 0.0000	1.0065 0.0000	0.4049 0.0000	0.6259 0.0000
KAL	0.6757 0.0000	1.2425 0.0000	0.9591 0.0000	0.4359 0.0000	-	1.0463 0.0000	2.3760 0.0000	1.2859 2044.2911	0.5885 0.0000	1.9179 0.0000
BKL	0.6713 0.0000	1.0366 968.7390	1.0863 0.0000	0.2171 0.0000	0.4147 0.0000	-	1.1551 0.0000	0.1481 0.0000	1.3527 336.2823	1.2143 0.0000
CHE	1.0275 0.0000	1.1594 0.0000	0.8218 0.0000	0.9686 412.9380	1.6874 0.0000	0.5870 0.0000	-	1.4819 0.0000	0.4550 0.0000	0.5724 0.0000
SND	0.7351 0.0000	0.6908 0.0000	0.8354 3990.1702	1.2364 2585.0881	0.5457 0.0000	1.3584 0.0000	1.3254 0.0000	-	0.2562 0.0000	1.0693 0.0000
DOU	0.7422 1207.0498	0.6883 0.0000	0.4183 190.5884	1.1336 0.0000	1.2416 31.7693	0.5938 0.0000	0.9717 0.0000	1.4846 0.0000	-	0.6207 0.0000
TAT	0.2560 0.0000	0.6350 0.0000	0.5017 0.0000	0.9218 0.0000	0.9933 0.0000	0.8295 0.0000	0.6043 0.0000	1.2800 0.0000	0.9626 3434.6332	-

**Table 5:** MANOVA results of the individual FCA scores in the range of 1–10.

	df	Pillai	app. F	pr
pop	9	1.93656	3.4271	<2 × 10^−16^ ***
coat color	1	0.03860	0.4697	0.9066
mtCR-1GL	1	0.07133	0.8986	0.5369

pop—local population, coat color type 1—4, mtCR-1GL—GENELAND population of mtCR-1 haplotype, df—degree of freedom, Pillai—Pillai statistics, app. F—approximate F value, pr—probability, and ***—significance.

**Table 6:** Measures of genetic diversity in the Tunisian hare populations and two GENELAND populations (genetic clusters) based on mtDNA sequences.

	Populations	h	π	k
Local Populations	BKL (26)	0.628	0.00984	4.526
	CHE (8)	0.893	0.01219	5.607
	NAD (8)	0.436	0.01802	8.291
	KAL (06)	0.867	0.01580	7.267
	WES (05)	0.900	0.02832	13.000
	BEJ (16)	0.983	0.02869	13.167
	SND (21)	0.757	0.01511	6.933
	STH (08)	0.821	0.01685	7.750
	DOU (15)	0.914	0.02789	12.800
	TAT (22)	0.962	0.03321	15.276
GENELAND	population 1 (77)	0.906	0.01342	6.162
	population 2 (61)	0.981	0.02820	12.942
Total		0.966	0.02735	12.55198

h, haplotypic diversity; π, nucleotide diversity; k, mean number of pairwise differences; values in parentheses indicate sample size.

## Data Availability

Sequence data are downloadable by respective accession numbers from the NCBI web page.

## Abbreviations

The following abbreviations are used in this manuscript:

Mt	Mitochondrial
DNA	Deoxyribonucleic acid
OXPHOS	Oxidative phosphorylation
